# Suppression of Th1-Mediated Autoimmunity by Embryonic Stem Cell-Derived Dendritic Cells

**DOI:** 10.1371/journal.pone.0115198

**Published:** 2014-12-18

**Authors:** Tokunori Ikeda, Shinya Hirata, Koutaro Takamatsu, Miwa Haruta, Hirotake Tsukamoto, Takaaki Ito, Makoto Uchino, Yukio Ando, Seiho Nagafuchi, Yasuharu Nishimura, Satoru Senju

**Affiliations:** 1 Department of Immunogenetics, Kumamoto University Graduate School of Medical Sciences, Kumamoto, Japan; 2 Japan Science and Technology Agency, CREST, Kawaguchi, Japan; 3 Department of Hematology, Kumamoto University Graduate School of Medical Sciences, Kumamoto, Japan; 4 Department of Neurology, Kumamoto University Graduate School of Medical Sciences, Kumamoto, Japan; 5 Department of Pathology and Experimental Medicine, Kumamoto University Graduate School of Medical Sciences, Kumamoto, Japan; 6 Jonan Hospital, Kumamoto, Japan; 7 Department of Medical Science and Technology, Kyushu University, Graduate School of Medical Sciences, Fukuoka, Japan; University Hospital of Heidelberg, Germany

## Abstract

We herein demonstrate the immune-regulatory effect of embryonic stem cell-derived dendritic cells (ES-DCs) using two models of autoimmune disease, namely non-obese diabetic (NOD) mice and experimental autoimmune encephalomyelitis (EAE). Treatment of pre-diabetic NOD mice with ES-DCs exerted almost complete suppression of diabetes development during the observation period for more than 40 weeks. The prevention of diabetes by ES-DCs was accompanied with significant reduction of insulitis and decreased number of Th1 and Th17 cells in the spleen. Development of EAE was also inhibited by the treatment with ES-DCs, and the therapeutic effect was obtained even if ES-DCs were administrated after the onset of clinical symptoms. Treatment of EAE-induced mice with ES-DCs reduced the infiltration of inflammatory cells into the spinal cord and suppressed the T cell response to the myelin antigen. Importantly, the ES-DC treatment did not affect T cell response to an exogenous antigen. As the mechanisms underlying the reduction of the number of infiltrating Th1 cells, we observed the inhibition of differentiation and proliferation of Th1 cells by ES-DCs. Furthermore, the expression of VLA-4α on Th1 cells was significantly inhibited by ES-DCs. Considering the recent advances in human induced pluripotent stem cell-related technologies, these results suggest a clinical application for pluripotent stem cell-derived dendritic cells as a therapy for T cell-mediated autoimmune diseases.

## Introduction

Autoimmune diseases occur and develop when immunological self-tolerance is broken by some mechanisms and autoreactive lymphocytes attack tissues [1]. Although therapeutic drugs including corticosteroids, other immune suppressants, and molecularly targeted drugs are effectively used for the treatment of some autoimmune diseases, long-term administration of these drugs increases the risk of systemic immune suppression and consequent opportunistic infections or the development of cancer [2], [3]. Therefore, it would be greatly advantageous if we could develop a therapeutic means of inhibiting autoimmunity while preserving immunity against exogenous pathogens.

Dendritic cells (DCs) are professional antigen-presenting cells with the ability to stimulate naïve T cells and initiate primary immune responses [4]. They are also involved in the maintenance of peripheral self-tolerance by promoting the function of regulatory T cells (Treg) or by inhibiting activation of auto-reactive T cells [5]–[8]. Moreover, a DC subset was reported to contribute to the polarizing influences on T helper differentiation [9]–[11]. The DC function can be altered by some immune suppressive drugs, and this mechanism has been shown to play a role in the control of autoimmune diseases [12], [13]. For these reasons, therapies utilizing DCs have previously been attempted for autoimmune diseases. Indeed some animal models of autoimmune diseases were prevented by the transfer of modulated DCs [14]–[18].

Our laboratory previously investigated the treatment of autoimmune diseases by DCs. We have established methods to generate DCs *in vitro* from embryonic stem (ES) and induced pluripotent stem (iPS) cells, which are characterized by pluripotency and an infinite propagation capacity [19]–[21]. Moreover, we also established a strategy for the genetic modification of ES or iPS cell-derived DCs (ES-or iPS-DCs) [19]–[22] in which a gene expression vector is introduced into ES or iPS cells that are then induced to differentiate into ES- or iPS-DCs. This enabled us to demonstrate that genetically modified ES- or iPS-DCs exert preventive or therapeutic effects against mouse models of autoimmunity or cancer [20]–[23].

We previously carried out the intraperitoneal (i.p.) transfer of genetically modified ES-DCs to protect against myelin oligodendrocyte glycoprotein (MOG)-induced experimental autoimmune encephalomyelitis (EAE). The ES-DCs presented MOG peptide in the context of MHC class II molecules and expressed an immunoregulatory molecule, TNF-related apoptosis-inducing ligand (TRAIL) or Programmed cell death 1 ligand 1 (PD-L1) [22]. Although this treatment is considered a promising means to regulate responses in an antigen-specific manner, multiple auto-antigens are involved in the pathogenesis of most human autoimmune disorders. Thus, it should be proved that the treatment is effective not only against single antigen-induced autoimmunity, but also spontaneously occurring autoimmunity involving multiple auto-antigens.

In the present study, therefore, we examined the protective effect of non-genetically modified and genetically modified ES-DCs against diabetes in nonobese diabetic (NOD) mice, which is a spontaneously occurring autoimmune disease involving multiple auto-antigens. Both ES-DC subsets prevented the onset of diabetes. Furthermore, the intravenous injection of ES-DCs exhibited a therapeutic effect against MOG-induced EAE. ES-DCs suppressed the differentiation and proliferation of Th1 cells both *in vitro* and *in vivo*. These results suggest that therapy with pluripotent stem cell-derived DCs is a novel and rational approach for the treatment of autoimmune diseases.

## Material and Methods

### Mice

C57BL/6 wild-type (B6) mice were purchased from the Clea Animal Cooperation (Tokyo, Japan), B6.SJL-ptprc(a)Pep3(b)BoyJ (CD45.1^+^) mice from the Jackson Laboratory (Bar Harbor, ME, USA), and nonobese diabetic/severe combined immunodeficient (NOD/SCID) mice from Charles River Laboratories Japan (Yokohama, Japan). Ovalbumin (OVA)-specific I-A^b^-restricted OT-II T cell receptor (TCR)–transgenic mice were kindly provided by S. Koyasu (Keio University, Tokyo, Japan), and NOD mice by K. Yokono (Kobe University, Kobe, Japan). All the experimental procedures were carried out in accordance with guidelines of Center for Animal Resources and Development in Kumamoto University. The protocol was approved by the Institutional Animal Committee of Kumamoto University (Permit Number: B24-147). All efforts were made to minimize the number of mice and their suffering, and mice were euthanized with isoflurane. All experiments were performed in accordance with the ARRIVE guidelines [24].

### Peptides and cell lines

Mouse MOG peptide 35–55 (MEVGWYRSPFSRVVHLYRNGK) and OT-II peptide (chicken OVA peptide 323–339: ISQAVHAAHAEINEAGR) were purchased from AnyGen (Gwangju, Korea) and Peptide 2.0 Inc (Chantilly, VA, USA), respectively. Establishment and characterization of the NOD ES cell line was previously reported [25]. B6 ES cell line (BRC5) was purchased from the RIKEN BioResource Center (Tsukuba, Japan). These ES cell lines and the M-CSF-defective bone marrow-derived stromal cell line OP9 were maintained as described previously [19].

### Antibodies

The following antibodies (Abs) were used in this study: FcR blocking Ab (anti-mouse CD16/CD32; clone: 2.4G2, BD Pharmingen), fluorescein isothiocyanate (FITC)- and phycoerythrin (PE)-conjugated anti-mouse CD4 (Clone: RM4-5, eBioscience), PerCP-conjugated anti-mouse CD4 (Clone: RM4-5, BD Pharmingen), FITC-conjugated anti-mouse CD8a (Clone: 53–6.7, eBioscience), FITC-conjugated anti-CD3 (Clone: 145-2C11, BioLegend), (PE-conjugated anti-mouse CD80 (Clone: 16-10A1, eBioscience), FITC-conjugated anti-mouse CD11c (Clone: N418, eBioscience), PE-conjugated anti-mouse CD86 (Clone: PO3.1, eBioscience), FITC-conjugated anti-mouse F4/80 (Clone: BM8, eBioscience), FITC-conjugated anti-mouse CD14 (Clone: Sa2-8, eBioscience), FITC-conjugated anti-mouse H-2D^b^ (Clone: CTDb, CALTAG), FITC-conjugated anti-mouse H-2K^b^ (Clone: CTKb, CALTAG) FITC-conjugated anti-mouse H-2K^d^ (Clone: SF1-1.1, BD Pharmingen), FITC-conjugated anti-mouse I-A^b^ (Clone: 28-16-8S, CALTAG), FITC-conjugated anti-mouse I-A^k^ (Clone: 10-3.6, BioLegend), PE-conjugated anti-mouse TRAIL (Clone: N2B2, eBioscience), FITC-conjugated anti-human CD74 (Clone: M-B741, BD Pharmingen), FITC-conjugated anti-mouse/rat Foxp3 (Clone: FJK-16s, eBioscience), FITC-conjugated anti-mouse CD45.1 (Clone: A20, BioLegend), PerCP/Cy5.5-conjugated anti-mouse CD45.2 (Clone: 104, BioLegend), PE-conjugated anti-mouse CD11b (Clone: M1/70, BD Pharmingen), Anti-HA-Fluorescein (Clone: 3F10, Roche), PE-conjugated anti-mouse VLA-4α (Clone: 9C10, BioLegend), PE/Cy7-conjugated anti-mouse CD69 (Clone: H1.2F3, BioLegend), FITC-conjugated anti-bromodeoxyuridine (BrdU) (Clone: B44, BD Pharmingen), PE-conjugated anti-mouse IL-17A (Clone: eBio17B7, eBioscience), PerCP/Cy5.5-conjugated anti-mouse IFN-γ (Clone: XMG1.2, BioLegend), FITC- (BD Pharmingen) and PerCP/Cy5.5-conjugated (BioLegend) mouse IgG2a isotype-matched control, FITC- and PE-conjugated rat IgG2a isotype-matched control (eBioscience), FITC- and PE-conjugated Armenian hamster IgG (eBioscience), PE-conjugated rat IgG2b isotype-matched control (BD Pharmingen), FITC- and PE- and PerCP-conjugated rat IgG2a isotype-matched control (BD Pharmingen), PerCP/Cy5.5-conjugated rat IgG1 isotype-matched control (BioLegend), PE/Cy7-conjugated Armenian hamster IgG (BioLegend), anti-and FITC-conjugated mouse IgM isotype-matched control (eBioscience).

### Flow-cytometry analysis

Cells were incubated with FcR blocking Abs on ice for 15 min and were subsequently labeled with specific Abs at 4°C. In some experiments, intracellular cytokine analysis was performed as described previously [26]. Briefly, cells were resuspended in RPMI 1640 medium supplemented with 10% fetal bovine serum and 2-ME. Phorbol 12-myristate 13-acetate (PMA; 50 ng/mL), ionomycin (500 ng/mL), and brefeldin A (10 µg/mL; all Sigma-Aldrich, St Louis, MO, USA) were added to the cells. After incubation for 5 h, cells were washed and stained for surface markers, and subsequently fixed and permeabilized using IntraPrep reagent (Beckman Coulter) according to the manufacturer's instructions, followed by intracellular staining for IL-17A and IFN-γ. To demonstrate the specificity of staining, isotype-matched control mAb were also used. The stained cells were analyzed using FACS Calibur (BD Biosciences). Data were analyzed using FlowJo software (Treestar).

### Plasmid construction and transfection into ES cells

To generate an insulin peptide-presenting vector, double-stranded oligo DNA encoding the insulin p9-23 epitope, 5′-tcccacctggtggaggctctctacctggtgtgttgtggggagcgtggc-3′, was inserted into the previously reported human invariant (Ii)-based epitope-presenting vector, pCI30 [19], [22]. The coding region of this construct was transferred into pCAG-IPuro, a mammalian expression vector driven by a CAG promoter and containing the internal ribosomal entry site (IRES)-neomycin resistance gene cassette, to generate pCAG-Insulin-IPuro. cDNA encoding mouse glutamic acid decarboxylase (GAD65) was prepared by PCR using the pCMV-SPORT6 plasmid vector containing mouse GAD65 (Life Technologies) as a template. To generate cDNA encoding GAD65 493–586 linked with a hemagglutinin tag (HA: YPYDVPDYA) at the C-terminus, PCR was carried out using the forward primer, 5′-CACCTCGAGATGGTGTTTGATGGGAAGCCTC-3′ and reverse primer, 5′-TTATTAAGCGTAGTCTGGGACGTCGTATGGGTACAAATCTTGTCCGAGGCGTTC-3′. The GAD65-HA cDNA was subcloned into pENTR/D-Topo (Life Technologies). To amplify the cDNA fragment of human Ii chain linked to nucleotide sequences for *Xho* I at the C-terminus, pCI30 vector was PCR amplified using the forward primer, 5′-CACCATGGATGACCAGCGCGACCTTATCTCC-3′ and reverse primer, 5′-AACTCGAGAAGCTTCATGCGCAGGTTCTCCAG. The human Ii chain fragment cDNA was subcloned into pENTR/D-Topo, and the vector was digested with *Not* I and *Xho* I. The GAD65-HA vector was also digested with these enzymes, and the isolated Ii chain fragment cDNA was subcloned into the GAD65-HA vector. This fused DNA fragment was subsequently transferred into pCAG-IPuro to generate pCAG-GAD65-IPuro. The pCAG-TRAIL-INeo vector was generated previously [22]. These plasmid vectors were introduced into ES cells, and genetically modified ES cells were differentiated into ES-DCs as described previously [19], [22]. Surface markers on differentiated ES-DCs were checked by flow cytometry (S1A–B Figure).

### Cytotoxicity assay

ES-DCs or genetically modified ES-DCs were co-cultured with L929 cells as target cells (5×10^3^/well) at the indicated effector-to-target ratio, and a standard ^51^Cr-release assay was performed as described previously [27].

### Induction of EAE

EAE was induced in 6- to 8-week old female B6 mice by subcutaneous immunization with 200 µg of MOG 35–55 peptide emulsified in CFA containing 2 mg/mL of heat-killed *Mycobacterium tuberculosis* H37RA (Difco Laboratories) at the base of the tail on day 0, as described previously [26]. Additionally, mice received 350 ng of *Bordetella pertussis* toxin (Calbiochem) i.p. in 0.5 ml of PBS on days 0 and 2. Clinical signs of EAE were assessed according to the following score: 0, normal; 1, weakness of the tail and/or paralysis of the distal half of the tail; 2, loss of tail tonicity and abnormal gait; 3, partial hindlimb paralysis; 4, complete hindlimb paralysis; 5, forelimb paralysis or moribundity; 6, death.

### Treatment of mice with ES-DCs

Four-week old prediabetic female NOD mice were administered i.p. with ES-DCs (5×10^5^ cells/injection/mouse) once every 2 weeks and the treatment was repeated one to five times. Control NOD mice were injected with PBS. PBS- or ES-DC-treated NOD mice were observed until the age of 40 weeks or more. Blood samples were obtained from the tail vein to determine blood glucose levels with a glucometer (Arkray). Mice were considered diabetic when blood glucose levels were above 300 mg/dL. B6 or CD45.1^+^ mice were injected i.v. with 1−2×10^6^ undifferentiated ES cells, ES-DCs per mouse or PBS for control at day 10 after EAE-induction, and the mice were observed over a period of 30 days. In some experiments, mice were injected i.p. with 1 mg of BrdU (BD Pharmingen) daily for 5 days from the day of ES-DCs or PBS injection.

### Adoptive cell transfers into NOD/SCID mice

5×10^6^ splenocytes from diabetic NOD mice were intravenously (i.v.) injected into 6-week-old female NOD/SCID mice, and NOD/SCID mice were observed for the onset of diabetes by measuring blood glucose levels. After the next day of confirming the onset of diabetes, diabetic NOD/SCID mice were administered i.p. with ES-DCs (5×10^5^ cells/injection/mouse) or PBS once every 3 days, total 3 times, and blood glucose levels was monitored.

### Preparation of tissue-infiltrating mononuclear cells

In EAE-induced mice, mononuclear cells infiltrating the liver, lung, and spinal cord were isolated as described previously on days 5, 9, or 12 after i.v. injection with ES-DCs [28], [29]. In brief, mice were perfused through the left cardiac ventricle with cold 1 mM EDTA/PBS. The collected organs were cut into pieces and digested with 2.5 mg/mL collagenase D (Roche Diagnostics) and 1 mg/mL DNase I (Sigma) at 37°C for 45 min. The digested organs were filtered through a cell strainer (70 µm) and centrifuged. The pellets were suspended in 30% Percoll and overlaid on the top of a gradient containing 37% or 70% Percoll solution. The gradient was centrifuged and mononuclear cells were collected from the 37–70% interface.

### Helper Th cell differentiation

Naïve CD4^+^ CD62L^+^ T cells from the spleens of unprimed OT-II TCR transgenic mice were purified using the MACS cell sorting system (Miltenyi Biotec). CD4^+^ CD62L^+^ T cells (7.0×10^5^) were stimulated with 5 µg/mL plate-bound anti-CD3 Abs (BD Pharmingen) and 1 µg/ml soluble anti-CD28 Abs (BD Pharmingen) for three days in RPMI-1640 supplemented with 10% fetal bovine serum and 2-ME in the presence of recombinant cytokines. T cells were polarized with 10 ng/mL recombinant mouse IL-12 (R&D Systems), 5 ng/ml mouse IFN-γ (R&D Systems), 20 U/mL human IL-2, and 5 µg/mL anti-IL-4 (BD Pharmingen) for Th1 cell differentiation, 5 ng/mL human TGF-β1 (PeproTech), 20 ng/mL mouse IL-6 (R&D Systems), 20 U/mL human IL-2, and anti-IL-4 and anti- IFN-γ (2 µg/mL; BD Pharmingen) for Th17 cell differentiation, as well as 5.0 ng/mL human TGF-β1 (PeproTech), 100 U/mL human IL-2, anti-IL-4, and anti- IFN-γ (5 µg/mL; BD Pharmingen) for induced Treg (iTreg) cell differentiation.

### Generation of bone marrow-derived dendritic cells (BM-DCs)

The generation of BM-DCs was carried out as described previously [19]. In brief, BM cells from B6 mice were cultured in RPMI-1640 supplemented with 10% fetal bovine serum, GM-CSF (500 U/mL) and 2-ME for 12 days.

### T cell-proliferation assay

Twenty-one days after immunization with MOG peptide or 50 µg KLH (Sigma), inguinal lymph node (ILN) cells were isolated and cultured (2.0×10^5^ cells/well) with MOG peptide (0, 1, 3.2 µM) or KLH protein (0, 0.1, 1, 10, 100 µg/mL) in 96-well flat-bottomed plates for three days. In some experiments, differentiated Th1-, Th17- or iTreg cells (2.5×10^4^ cells) from OT-II TCR transgenic mice were co-cultured with X-ray irradiated (45 Gy) BM-DCs (1.0×10^4^ cells) in the presence of OT-II peptide (2.5 µM) and 20 U/mL human IL-2 in 96-well plates for 3 days. ES-DCs were added at increasing doses. [^3^H]-thymidine (248 GBq/mM) was added to the culture (37 kBq/well) for the final 12 h. At the end of the culture, cells were harvested onto glass fiber filters (Wallac) and the incorporation of [^3^H]-thymidine was measured by scintillation counting.

### Immunohistochemical analysis

The pancreas and spinal cords were removed from mice and fixed in formalin, embedded in paraffin, sectioned, and stained with H&E. In the tissue sections of the pancreas, a minimum of 30 islets were counted from each NOD mouse, and insulitis was scored as follows: 0, normal; 1, peri-insulitis; 2, leukocytic infiltration of up to 50%; 3, leukocytic infiltration over 50%.

### Statistical analysis

Kaplan-Meier log-rank analysis was used for survival data and unpaired one-way ANOVA with Scheffe's post-hoc tests, student's *t* test and Mann-Whitney U test were used to determine statistically significant differences. A value of *p*<0.05 was considered statistically significant.

## Results

### Inhibition of diabetes in NOD mice by ES-DCs with or without transgene-derived immunoregulatory molecules

We previously demonstrated that MOG-induced EAE was prevented by the i.p. transfer of genetically modified ES-DCs, which presented MOG peptides in the context of MHC class II molecules and expressed the immunoregulatory molecule TRAIL [22]. However, as various auto-antigens are etiologically involved in human autoimmunity, we investigated the preventive effects of genetically modified ES-DCs in another autoimmune model: NOD mice. NOD mice are widely used as an animal model for type 1 diabetes, and spontaneously develop diabetes as a result of the destruction of pancreatic β cells by auto-reactive T cells [30], [31]. Several molecules including insulin B chain, GAD65, GAD67, and heat shock protein 60 (HSP60) have been identified as the target auto-antigens in this disease [32]–[37]. We chose insulin B chain (peptides B9–23) and GAD65 (peptides 493–586) as auto-antigens for transfecting ES cells. It was previously reported that most CD4^+^ T cells infiltrating islets recognize insulin B chain 9–23 peptides [38], while, in the case of GAD65, two peptides (509–528 and 524–543) were shown to trigger the earliest response in NOD mice [37]. Therefore, GAD65 peptide was configured to include these epitopes prior to ES cell transfection.

For the efficient presentation of insulin or GAD65 peptide in the context of MHC class II molecules, we used a previously devised expression vector in which cDNA for the human MHC class II-associated Ii chain was mutated to contain the objective antigenic peptide in the class II-associated Ii peptide (CLIP) region [39]. An epitope inserted in this vector is efficiently presented in the context of co-expressed MHC class II molecules [19], [22]. Using this method, we generated the gene expression vectors pCAG-Insulin-IPuro and pCAG-GAD65-IPuro (S2A Figure). In pCAG-Insulin-IPuro, cDNA for the human Ii chain was mutated to contain an oligo DNA encoding the B9–23 epitope in the CLIP region. In pCAG-GAD65-IPuro, an HA tag was inserted at the C terminus of GAD65 peptide 493–586 and fused with the human Ii chain. The pCAG-TRAIL-INeo vector was generated previously [22].

Resultant single- or double-transfectant NOD ES cell clones were produced and differentiated into ES-DCs, and the expression of transfected genes was confirmed by flow-cytometric analysis (S2B–C Figure). ES-DC cell lines expressing TRAIL were identified using TRAIL-sensitive L929 cells (S2D Figure). We established three types of transfected ES-DCs: those expressing insulin peptide plus TRAIL (ES-DC-TRAIL/insulin), those expressing GAD65 peptide plus TRAIL (ES-DC-TRAIL/GAD65), and those expressing TRAIL only (ES-DC-TRAIL).

**Figure 1:**
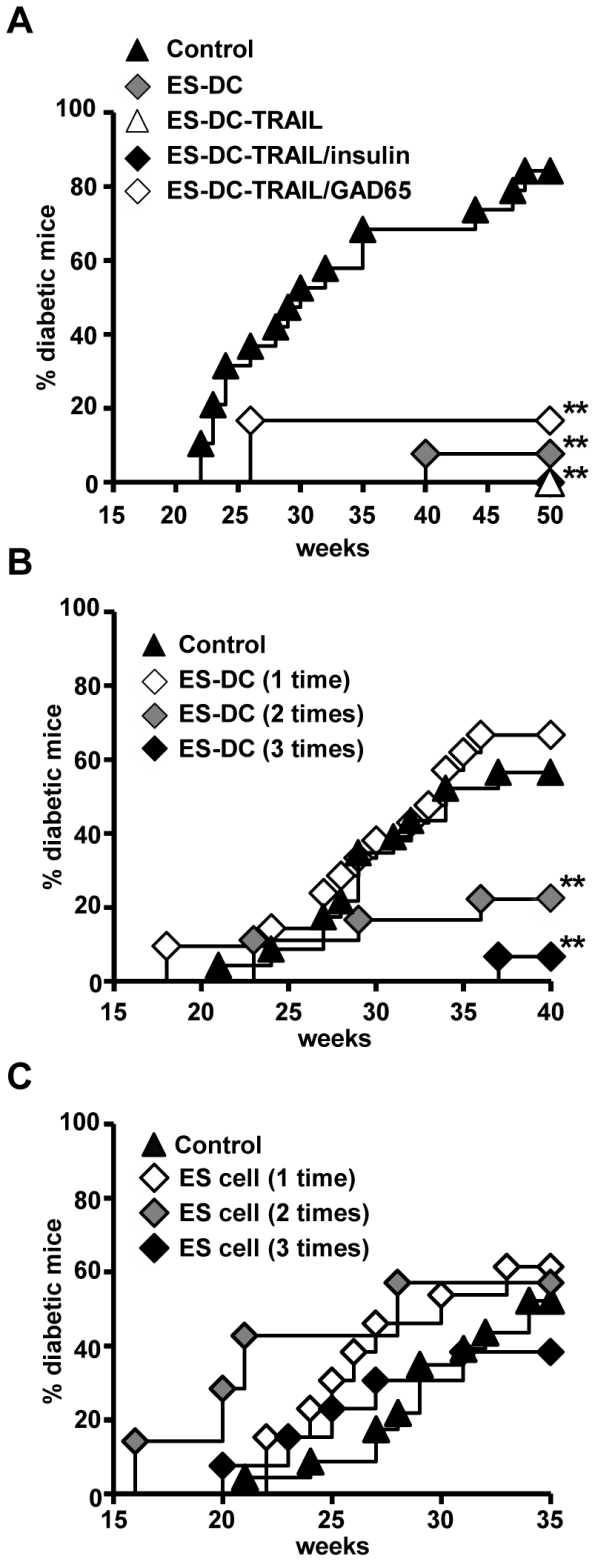
Prevention of diabetes development in NOD mice by ES-DCs but not ES cells. (A) 4-weeks old female prediabetic NOD mice were injected total 5 times i.p. with PBS (control), non-genetically modified ES-DCs (ES-DC), ES-DC-TRAIL, ES-DC-TRAIL/insulin, or ES-DC/GAD65 (5×10^5^ cells/injection/mouse) once in every two weeks, and then monitored for diabetes development (*n* = 6 per group). (B) ES-DCs were injected total 1 to 3 times as in (A) and monitored for diabetes development (*n* = 15−23 per group). (C) 4-weeks old female prediabetic NOD mice were injected 1 to 3 times i.p. with ES cells as in (A) and monitored for diabetes development (*n* = 7−13). Data are representative of two independent experiments. **, Diabetes development was significantly (*P*<0.01) suppressed as compared with the control mouse group based on Kaplan-Meier log-rank analysis.

Auto-reactive T cells to islet antigens are detectable in female prediabetic NOD mice from the age of 4 weeks [40]. To prevent the onset of diabetes, four week-old prediabetic female NOD mice were treated with five i.p. injections of each ES-DC line in a 2-week regimen. As expected, pretreatment with ES-DC-TRAIL/insulin and -TRAIL/GAD65 significantly inhibited the development of diabetes compared with non-treated control NOD mice, and ES-DC-TRAIL also suppressed the development of diabetes (Fig. 1A). In addition, non-genetically-modified ES-DCs also exhibited an equivalent disease prevention effect (Fig. 1A), suggesting that ES-DCs intrinsically possess a capacity to suppress autoimmune-mediated diabetes.

We determined to investigate the disease-preventive effect of non-genetically modified ES-DCs more precisely. As shown in Fig. 1B, the repeated administration of ES-DCs, but not a single dose, suppressed diabetes. Injection of undifferentiated NOD-ES cells never exerted disease-preventive effect irrespective of the number of times of injections. We thus ruled out the possibility that disease-preventive effect was mediated by undifferentiated ES cells contaminated in the ES-DC preparation (Fig. 1C). These results suggest that ES-DCs have an intrinsic function to suppress autoimmunity.

### Decreased IFN-γ-producing CD4^+^ T (Th1) and IL-17A-producing CD4^+^ T (Th17) cells in ES-DC-treated mice

**Figure 2:**
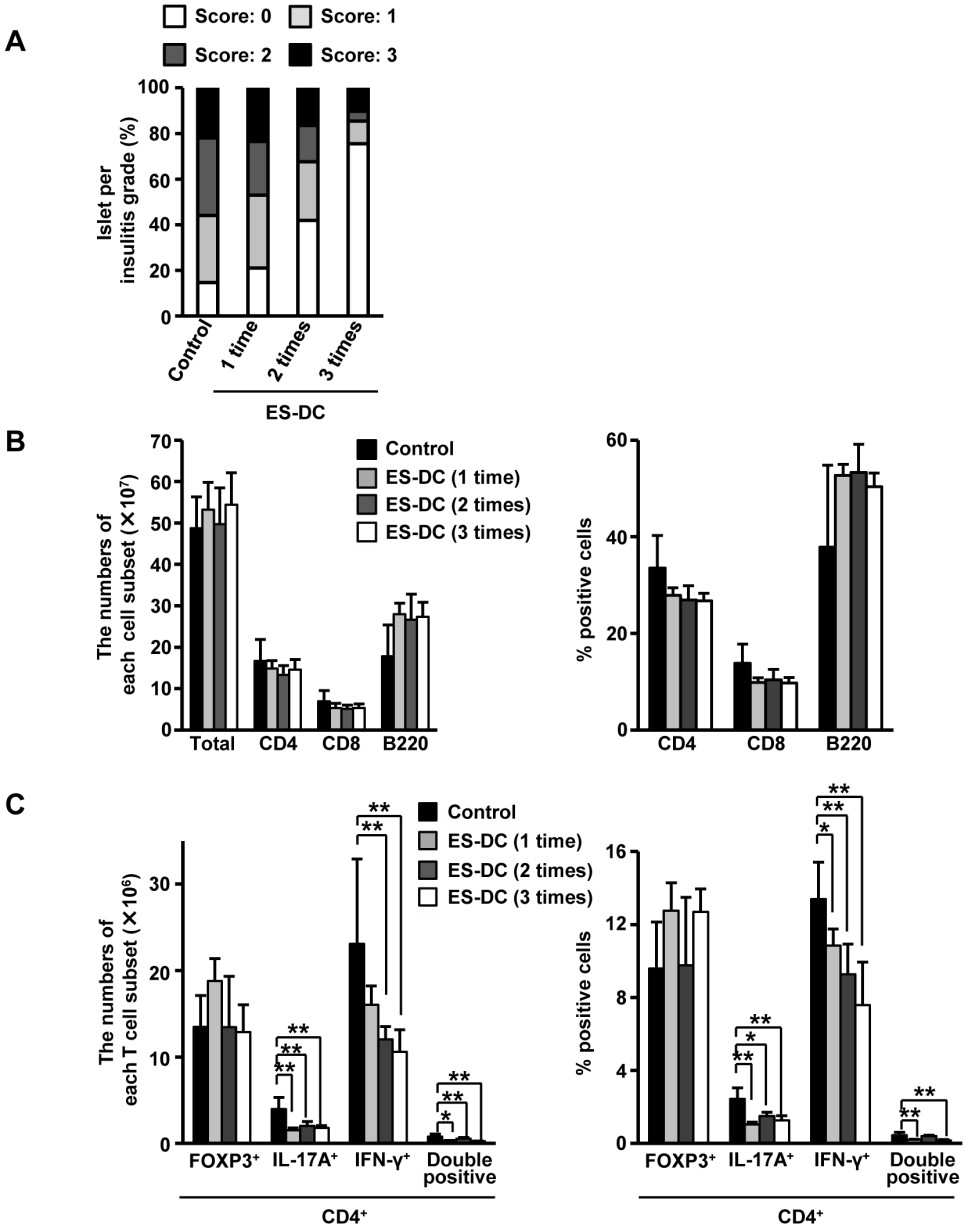
Decreased IFN-γ-producing CD4^+^ T (Th1) and IL-17A-producing CD4^+^ T (Th17) cells in ES-DC-treated NOD mice. (A) Histological sections of pancreata isolated from 18-weeks-old ES-DC-treated or control NOD mice were scored for insulitis as descried in *Material and Methods* (*n* = 5–6 per group). (B) The numbers (*left*) and frequencies (*right*) of total, CD4 ^+^, CD8^+^, or B220^+^ cells in the spleen from ES-DC-treated or control NOD mice were analyzed. (C) The numbers (*left*) and frequencies (*right*) of Foxp3^+^, IFN-γ^+^, IL-17A^+^, and IFN-γ^+^ IL-17A^+^ (double positive) CD4^+^ T cells within the total CD4^+^ T cells in the spleen from ES-DC-treated or control NOD mice were analyzed. Data (mean ± SD) are representative of two independent experiments. *P<0.05, **P<0.01, unpaired one-way ANOVA with Scheffe's post-hoc tests were used to determine statistically significant differences.

To assess whether ES-DC-induced diabetes resistance is associated with reduced islet inflammation, the development of insulitis was evaluated. Histopathology of the pancreas showed that ES-DC-treated NOD mice exhibited a reduction in the degree and severity of insulitis, depending on the times of injections (Fig. 2A). The progression of diabetes in NOD mice is mostly caused by IFN-γ^+^ CD4^+^ T (Th1) cells [41], [42]. We therefore investigated whether ES-DCs decreased the number or frequency of Th1 cells in the spleen. As shown in Fig. 2B–C, the numbers of total, CD4^+^, CD8^+^, and CD4^+^ Foxp3^+^ T cells (Treg cells) and B cells in ES-DC-treated mice were similar to those of control mice. On the other hand, the numbers of Th1 cells in ES-DC-treated mice was significantly lower than in control mice, although this was dependent on the times of injections (Fig. 2C). Furthermore, the numbers of IL-17A^+^ CD4^+^ T (Th17) cells and IFN-γ^+^ IL-17A^+^ CD4^+^ T cells (double-positive cells) were lower in ES-DC-treated mice (Fig. 2C). We also investigated the frequencies of respective cells in the spleen. In the result, the frequencies of Th1, Th17 and double-positive cells but not CD4, CD8, B, and Treg cells in ES-DC-treated mice were significantly decreased in comparison to control mice (Fig. 2B–C). These results suggest that ES-DCs prevented the onset of autoimmune diseases through inhibiting Th1 and Th17 cells.

### Therapeutic effect of ES-DCs against diabetic NOD/SCID mice

The above work revealed a disease-preventive effect of ES-DCs without forced expression of immunosuppressive molecules in multiple auto-antigen-related and naturally occurring autoimmunity in NOD mice. This therapy may prevent the recurrence of autoimmune disease. However, in clinical medicine, it is not practical to administrate ES-DCs prophylactically before the initial onset of autoimmune diseases. Therefore, we validated the effect of ES-DCs in a therapeutic but not prophylactic setting. Some studies reported that therapeutic administration of anti-CD3, anti-IL-17, anti-IL-25 antibodies and proinsulin DNA improved hyperglycemia in diabetic NOD mice [43]–[47].

**Figure 3:**
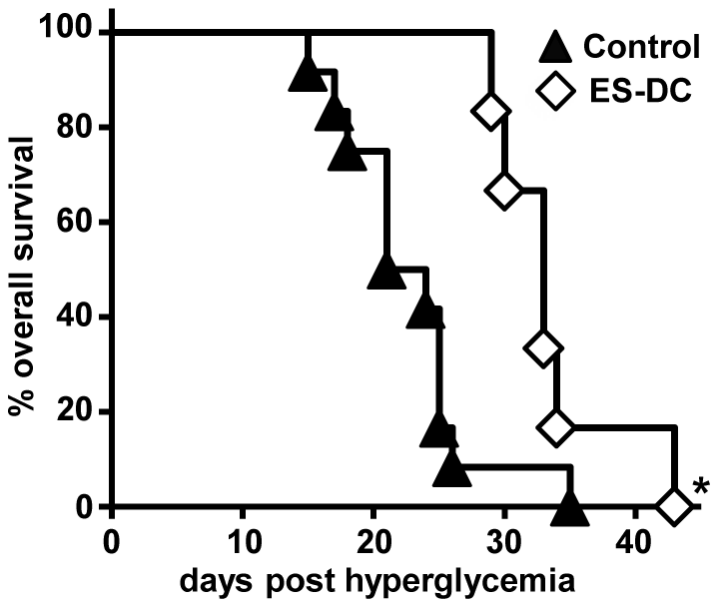
Improved survival duration in ES-DC-treated diabetic NOD/SCID mice. NOD/SCID mice were intravenously administrated with splenocytes (5×10^6^ cells/mice) from diabetic NOD mice, and checked the development of diabetes by measuring blood glucose levels. After the next day of confirming the onset of diabetes, diabetic NOD/SCID mice were administered i.p. with ES-DCs (5×10^5^ cells//mice) or PBS once every 3 days, total 3 times, and checked the survival rate of mice in each group (*n* = 6–12 per group). *P<0.05, statistical significance of the differences between each group were evaluated using Kaplan-Meier log-rank analysis.

To investigate therapeutic function of ES-DCs, splenocytes derived from diabetic NOD mice were i.v. injected into NOD/SCID mice. After the next day of the development of diabetes, diabetic NOD/SCID mice were treated with total three times i.p. injections of ES-DCs or PBS in every 3 days. In the result, although ES-DCs did not improve hyperglycemia in NOD/SCID mice (data not shown), ES-DC-treated NOD/SCID mice lived significantly longer than PBS-treated mice (Fig. 3). These results suggest the possibility that ES-DCs inhibited the further destruction of the islets, preserved the residual insulin-secreting function, and have a therapeutic effect.

### Therapeutic effect of ES-DCs against MOG-induced EAE

We next investigated the therapeutic effect of ES-DCs in a MOG-induced EAE model.

**Figure 4:**
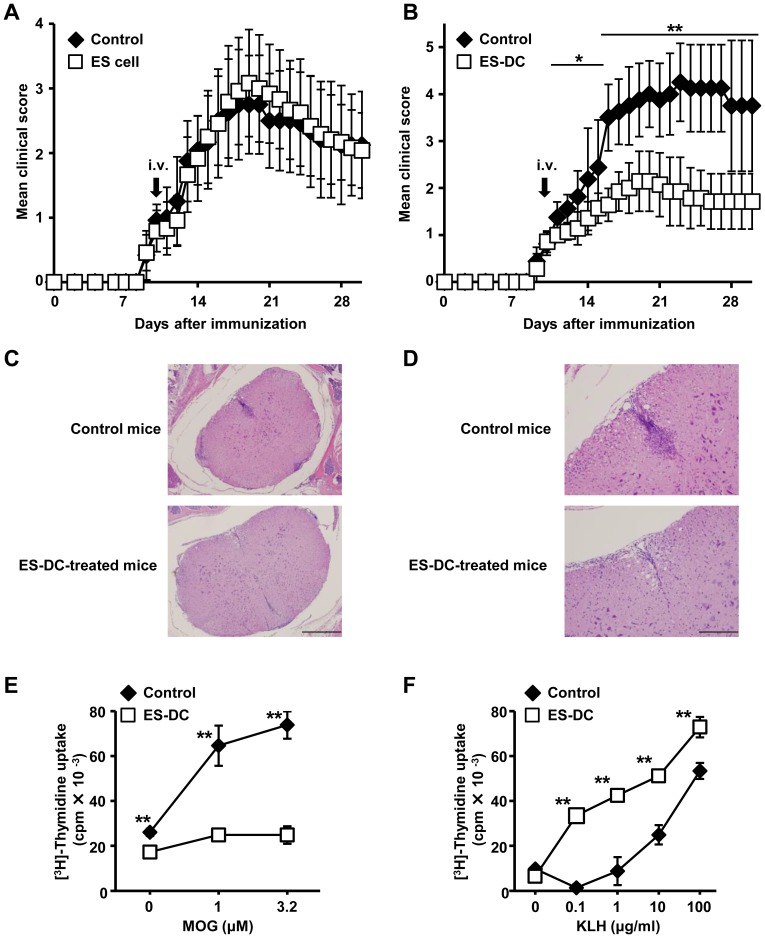
Therapeutic effect of ES-DCs in EAE-induced mice. EAE-induced B6 mice were administrated with undifferentiated ES cells or ES-DCs (2×10^6^ cells/mice) or PBS (control) at day 10 after immunization with MOG peptide. (A) Clinical scores in mice i.v. inoculated with ES cells and in control mice are shown (*n* = 12). Data (mean ± SD) are combined from a total of 2 separate experiments, the Mann-Whitney U test was used. (B) Clinical scores in mice i.v. inoculated with ES-DCs and in control mice are shown (*n* = 8). Data (mean ± SD) are representative of 2 independent experiments. *P<0.05, **P<0.01, the Mann-Whitney U test was used. (C–D) H&E staining of spinal cords isolated at day 18 after immunization from ES-DC-treated or control mice are shown. Lower (C) and higher (D) magnification views are shown and bars indicate 100 µm. (E) ILN cells (2.0×10^5^) were isolated from ES-DC-treated or control mice at 21 days after immunization, and cultured with MOG peptide (0, 1, 3.2 µM) for 3 days. The proliferative response was quantified by measuring [^3^H]-thymidine incorporation in the final 12 h of the culture. (F) Isolated ILN cells (2.0×10^5^) from ES-DC-treated or control mice 21 days after KLH-immunization were cultured with KLH protein (0, 0.1, 1, 10, 100 µg/ml) for 3 days. The proliferative response was quantified as (E). Representative data are shown as mean ± SD of triplicates from one of three independent experiments. **P<0.01, student's *t* test were used.

To induce EAE in B6 mice, we immunized the mice with the MOG 35–55 peptide, as described previously [26]. At first, we i.v. administrated undifferentiated B6-ES cells or ES-DCs on day 10 after the initial immunization, at which time the clinical symptoms of EAE had already established. We observed a significant alleviation of the severity of clinical symptoms following treatment with ES-DCs but not B6-ES cells (Fig. 4A–B). Histological examination revealed significantly lower infiltration of inflammatory cells into the spinal cord in ES-DC-treated mice compared with control mice (Fig. 4C–D).

We also analyzed the response of T cells isolated from EAE-induced mice to MOG peptide. Twenty-one days after immunization, ILN cells were isolated and cultured with MOG peptide. As shown in Fig. 4E, the proliferative response of these cells from ES-DC-treated mice was much lower than that of control mice. By contrast, the T cell response to the exogenous antigen, keyhole limpet hemocyanin (KLH), was unaffected by ES-DCs administration (Fig. 4F). Taken together, these data suggest that ES-DCs have both a preventive and therapeutic effect against autoimmune disease while preserving capacity of immune response against exogenous antigens.

### Decrease of Th1 cell infiltration into the target organ by ES-DCs treatment

**Figure 5:**
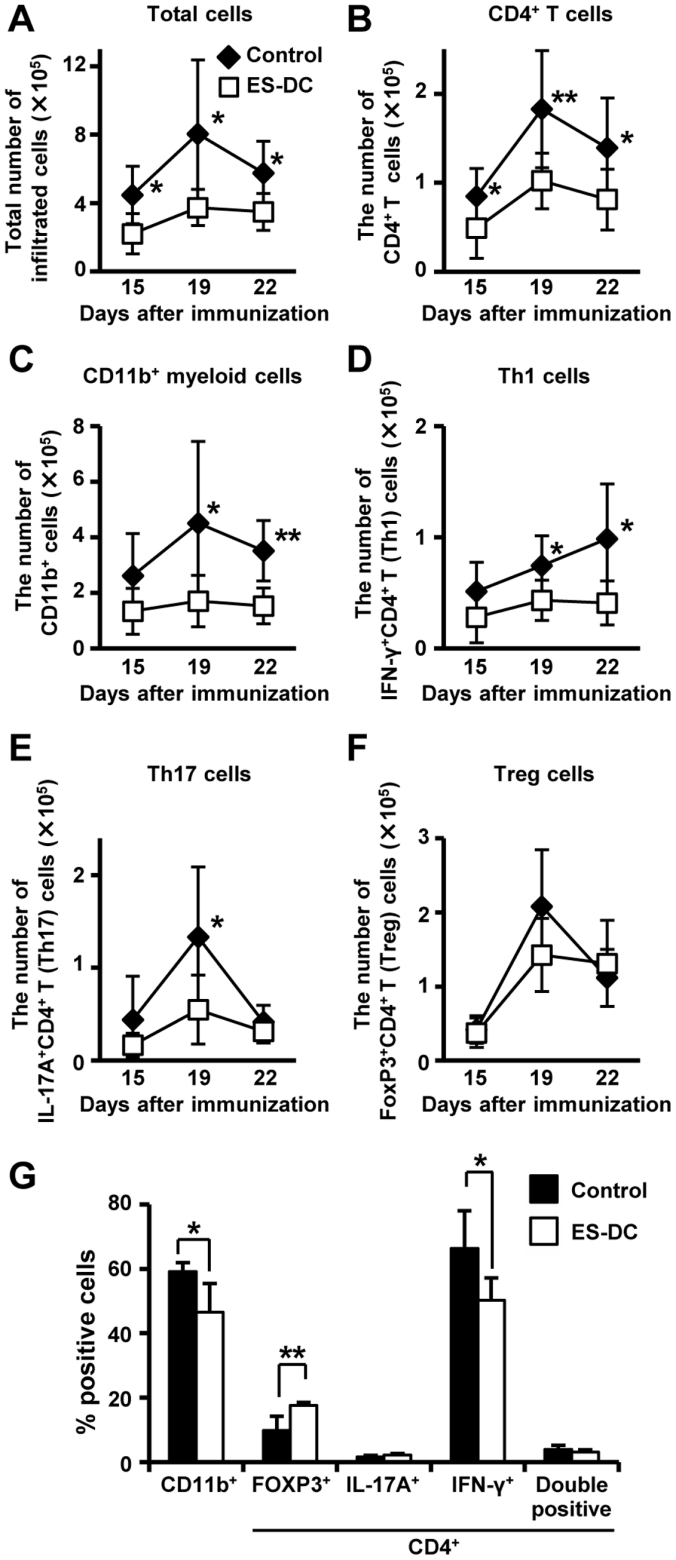
Decreased infiltration of Th1 cells and CD11b^+^ myeloid cells into spinal cord in ES-DC-treated mice. Spinal cord-infiltrating cells isolated from EAE-induced mice, treated subsequently with ES-DCs (2×10^6^ cells/mice) or untreated, were analyzed by flow cytometry to determine the numbers of infiltrating cells at day 15, 19, and 21 after immunization. (A) The total numbers of infiltrating cell into spinal cord in the two groups are shown (*n* = 5-7 per group). (B–F) The numbers of CD4^+^ T cells, CD11b^+^ myeloid cells, Th1, Th17, and Treg cells in the spinal cord are shown (*n* = 5–7 per group). (G) The frequencies of Foxp3^+^, IL-17A^+^, IFN-γ^+^, IFN-γ^+^ IL-17A^+^ (double positive) CD4^+^ T cells within the total CD4^+^ T cells and CD11b^+^ myeloid cells in the spinal cord on day 22 after EAE-induction (*n* = 5–7 per group). Data (mean ± SD) are representative of three independent experiments. *P<0.05, **P<0.01, student's *t* test were used.

As the prophylactic administration of ES-DCs affected the numbers and frequencies of Th1 and Th17 cells in NOD mice (Fig.2C), we hypothesized that the less severe symptoms in EAE-induced and ES-DC-treated mice was caused by the lower number or frequency of these cells infiltrating into the spinal cord. To test this possibility, we analyzed the number and frequency of infiltrating cells into the spinal cord on days 15, 19, and 22 in EAE-induced mice. The absolute numbers of infiltrating cells in ES-DC-treated mice were significantly decreased on days 15, 19, and 22 compared with control mice (Fig. 5A). The number of CD4^+^ T cells was also lower in all phases (Fig. 5B). Although the number of Th1 cells decreased on days 19 and 22 (Fig. 5D), there were no significant differences in the numbers of Treg, and double-positive cells between the two groups (Fig. 5F). In addition, the number of Th17 cells in ES-DC-treated mice decreased on day19 (Fig. 5E), and that of CD11b^+^ myeloid cells were decreased on days 19 and 22 in comparison to control mice (Fig. 5C). In the frequency of infiltrating cells, there were no significant differences in all subsets including Th1 and CD11b^+^ myeloid cells on days 15 and 19 (data not shown). On day 22, the frequencies of Th1 and CD11b^+^ myeloid cells in ES-DC-treated mice were lower, whereas that of Treg cells was higher than control mice (Fig. 5G). These results suggest that ES-DCs mainly function to inhibit the invasion of inflammatory cells such as Th1 and CD11b^+^ myeloid cells.

### ES-DCs inhibit the differentiation and proliferation of Th1 cells in vitro and in vivo

**Figure 6:**
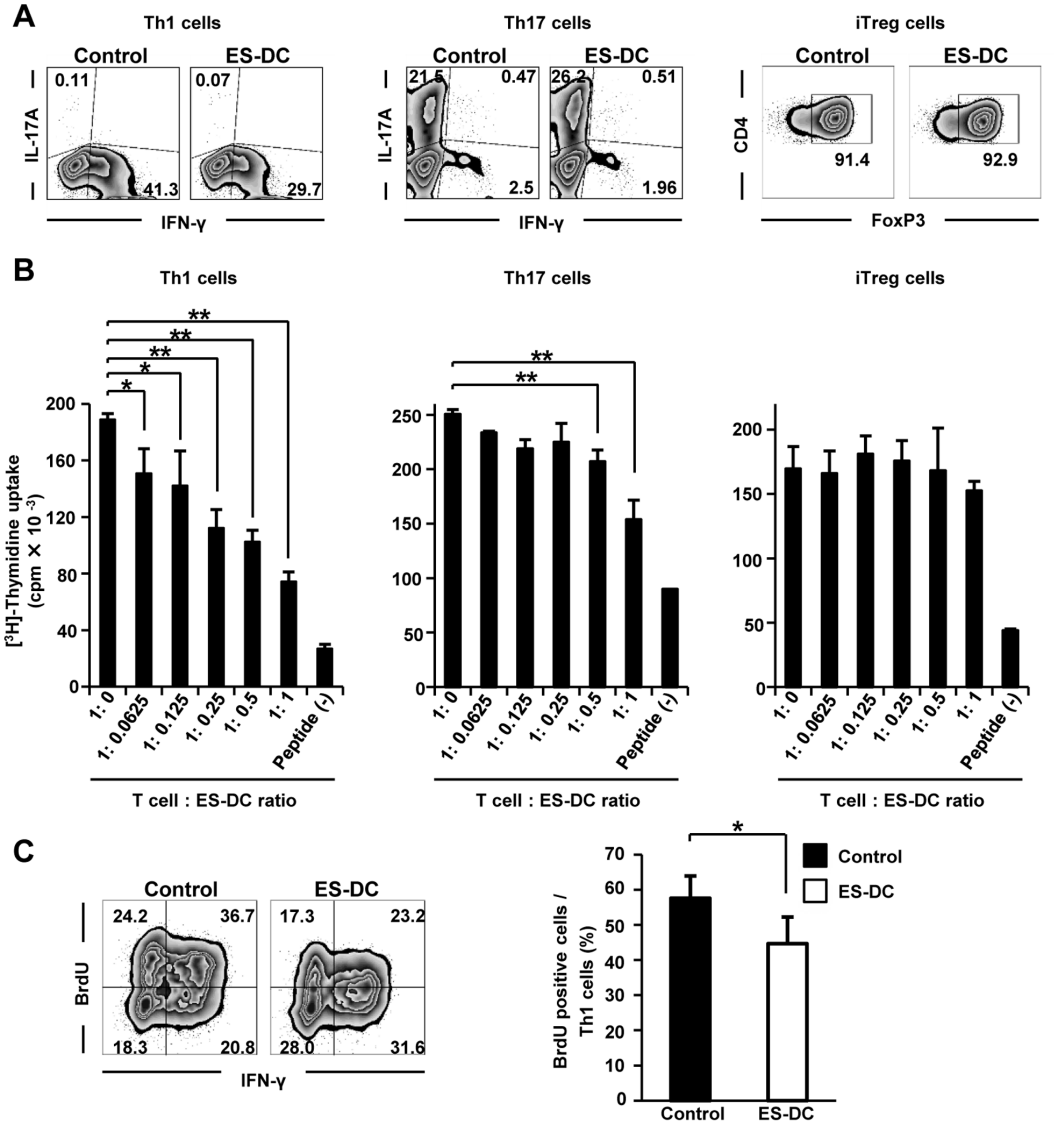
Regulation of the differentiation and proliferation of Th1 cells by ES-DCs. (A) 7.0×10^5^ naïve CD4^+^ T cells (CD4^+^ CD62L^+^) were purified from spleen of B6 mice and cultured for 3 days under Th1, Th17 and iTreg cell differentiation conditions as described in *Material and Methods*, in the presence or absence of cultured supernatant of ES-DCs. After culture, cells were stimulated with PMA/ionomycin, and analyzed for IL-17A and IFN-γ by intracellular staining. T cells cultured in iTreg-conditions were analyzed for FoxP3 by intracellular staining. (B) Differentiated OT-II Th1, Th17, and iTreg cells (2.5×10^4^) were cultured with irradiated BM-DCs (1.0×10^4^), OT-II peptide (2.5 µM) and increasing dosage of ES-DCs for 72 h. The proliferative response was quantified by measuring [^3^H]-thymidine incorporation in the final 12 h of the culture. (C) Mice were injected i.p. with 1 mg BrdU for consecutive 5 days from the starting date of the therapy with ES-DCs (2×10^6^) or PBS. BrdU incorporation into IFN-γ^+^ CD4^+^ T cells (Th1 cells) in the spinal cord of each mouse was assessed *ex vivo* after 5 days. Dot plots (*left*) and bar graphs (*right*) represent the frequencies of BrdU^+^ Th1 cells within the total CD4^+^ T cells and BrdU^+^ Th1 cells in Th1 cells, respectively (*n* = 5–6 per group). Data (mean ± SD) are representative of 3 or more independent experiments with similar results. *P<0.05; **P<0.01, student's *t* test were used.

As our *in vivo* observations suggested that ES-DCs negatively regulate Th1 cells, we next used *in vitro* Th cell differentiation cultures to determine whether ES-DCs impact on CD4^+^ effector T cell differentiation. Purified naïve CD4^+^ T cells (CD4^+^ CD62L^+^) from the spleen of naïve B6 mice were stimulated with anti-CD3 and anti-CD28 monoclonal antibodies and differentiated into Th1, Th17, or iTreg cells. To investigate the influence of ES-DCs on effector T cell differentiation, we cultured naïve CD4^+^ T cells in the cultured supernatant of ES-DCs. After 3 days, T cells were examined for IL-17, IFN-γ, and FoxP3 production. The cultured supernatant of ES-DCs strongly reduced the yield of Th1 cells, but had no effect on Th17 or iTreg cell differentiation (Fig. 6A). Next, to understand the influence of ES-DCs on the proliferation of differentiated T cells, OT-II Th1 or Th17 and iTreg cells were cultured with OT-II peptide, irradiated BM-DCs as antigen-presenting cells, and increasing doses of ES-DCs for 72 h. ES-DCs were found to reduce the proliferation of OT-II Th1 cells in a dose-dependent manner (Fig. 6B). Culture conditions with high doses of ES-DCs also suppressed Th17 but not iTreg cells (Fig. 6B). These results suggest that ES-DCs mainly down-modulate the proliferation of Th1 cells.

To explore this possibility *in vivo*, ES-DCs or PBS-injected mice were treated with BrdU for 5 days from day 10 after immunization, and BrdU incorporation into Th1 cells infiltrating into the spinal cord was measured by flow cytometry. We found that ES-DCs also impeded the proliferation of Th1 cells *in vivo* (Fig. 6C), confirming that they inhibited the differentiation and proliferation of Th1 cells.

### Immigration of ES-DCs into the spinal cord in EAE-induced mice

**Figure 7:**
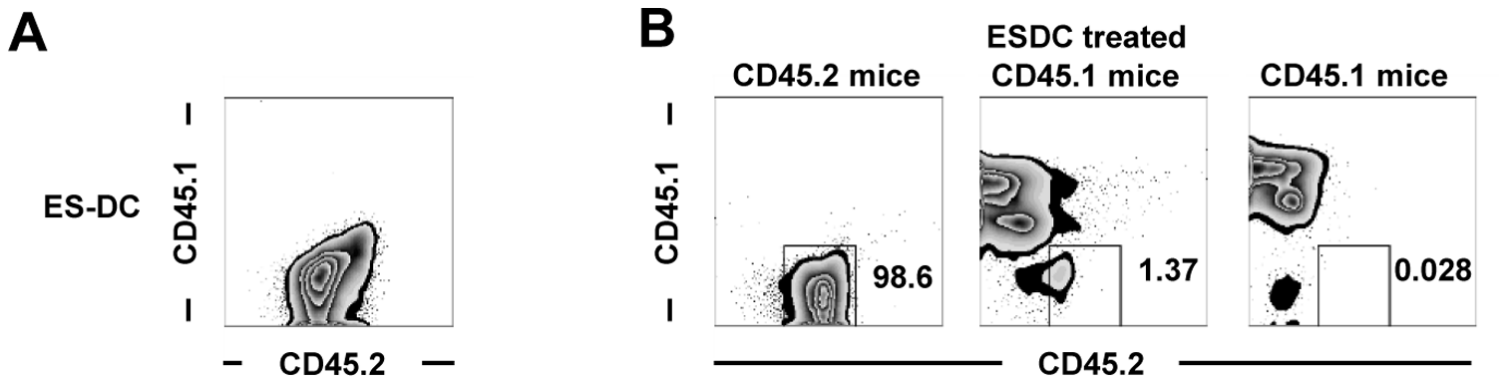
Migration of ES-DCs into the spinal cord. (A) The expression of CD45.1 and CD45.2 in ES-DCs was analyzed. (B) PBS or ES-DCs (2×10^6^) were i.v. injected to EAE-induced B6 (CD45.2^+^) or CD45.1^+^ mice. Ten days after the injection, isolated mononuclear cells in spinal cord were analyzed by flow cytometry to detect ES-DCs. Data are representative of 3 or more independent experiments with similar results.

Next, we investigated the distribution of *in vivo* administered ES-DCs. As ES-DCs are CD45.2 single-positive (Fig. 7A), once transferred into CD45.1^+^ mice they can readily be distinguished from intrinsic mouse cells. We i.v. injected ES-DCs into EAE-induced CD45.1^+^ mice, and 10 days later investigated the immigration of ES-DCs into spinal cord. In the results, ES-DCs were present in the spinal cord under EAE-induced conditions (Fig. 7B). These results suggest that ES-DCs immigrate into the inflamed organ and exert suppressive activity on Th1 cells.

### ES-DCs regulate the expression of VLA-4α in lungs Th1 cells

**Figure 8:**
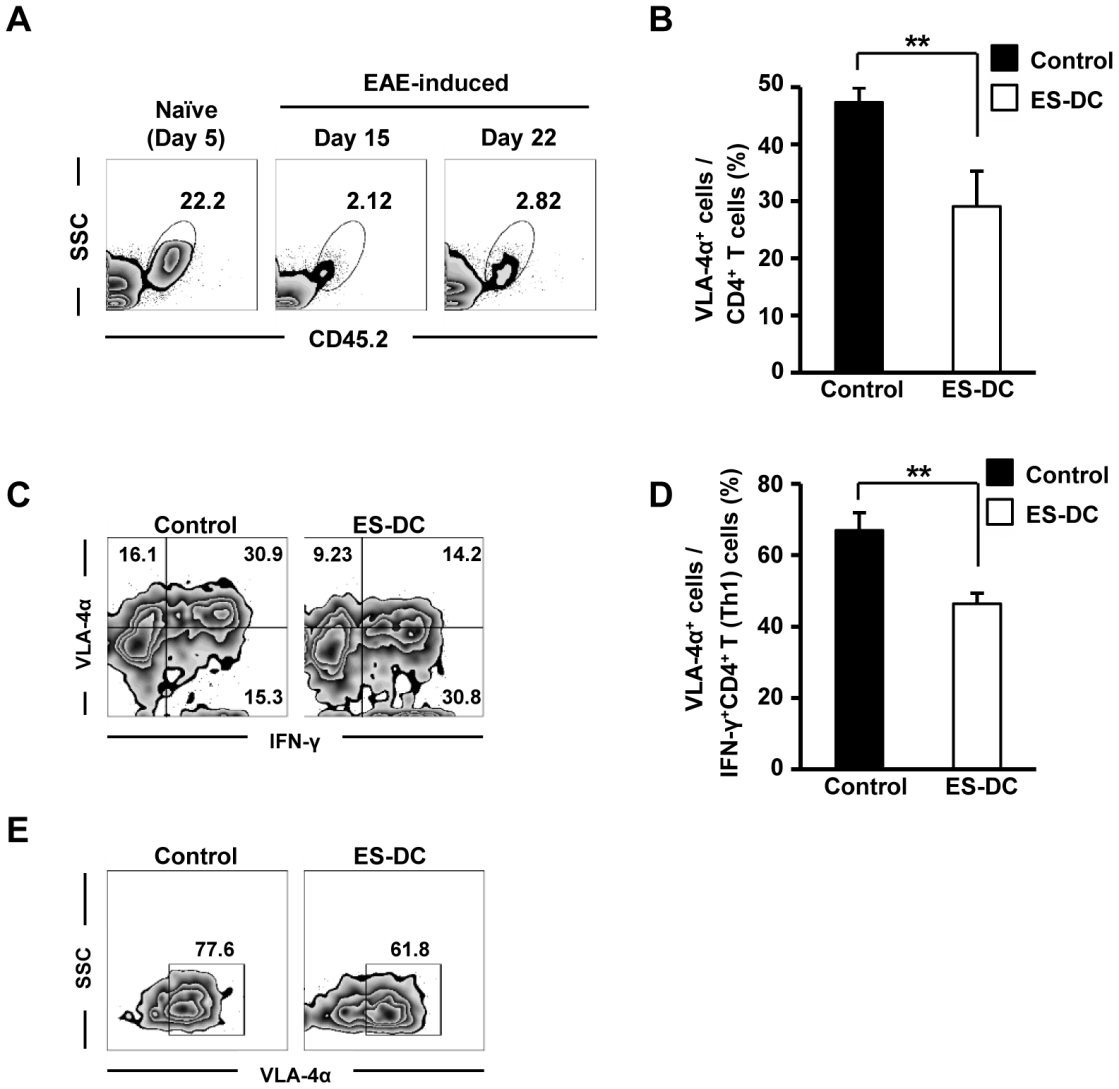
ES-DCs regulate the expression of VLA-4α^+^ on Th1 cells. (A) ES-DCs (1×10^6^) were intravenously administrated to naïve or EAE-induced CD45.1^+^ mice. 5 or 12 days after the administration, the distribution of ES-DCs in the lungs was investigated by flow cytometry. (B–D) EAE-induced B6 mice were administrated ES-DCs (2×10^6^) or PBS (control) at day 10 after immunization. 5 days after the injection of ES-DCs, the infiltrating cells in the lung were analyzed (*n* = 5). (B) The frequency of VLA-4α^+^ cells in CD4^+^ T cells were determined. (C) Representative data showing the expression of VLA-4α^+^ and IFN-γ^+^ in CD4^+^ T cells are shown. (D) The Frequency of VLA-4α^+^ IFN-γ^+^ CD4^+^ T cells in IFN-γ^+^ CD4^+^ T (Th1) cells. (E) Th1-differentiated OT-II T cells (2.5×10^4^) were cultured with irradiated BM-DCs (1.0×10^4^), OT-II peptide (2.5 µM) and ES-DCs (1.5×10^3^) *in vitro*. 3 days after, VLA-4α^+^ cells in CD3^+^ CD69^+^ T cells were investigated. Data (mean ± SD) are representative of three independent experiments. **P<0.01, student's *t* test were used.

Recently, it has been reported that the lungs are key for auto-reactive T cells to induce the expression of some chemokine receptors and adhesion molecules to enable entry into target organs [48]. As ES-DCs were localized in the lungs (Fig. 8A), we considered the possibility that ES-DCs affect the expression of homing molecules on auto-reactive T cells in the lung, and focused on integrin α4β1 (very late antigen-4, VLA-4α). VLA-4α controls the migration of lymphocytes into affected organs by binding with the adhesion molecule VCAM-1, which is expressed on endothelial cells [49], [50]. Five days after the injection of ES-DCs, the frequency of VLA-4α^+^ CD4^+^ T cells in ES-DC-treated mice was lower than in untreated mice (Fig. 8B). Moreover, the proportion of VLA-4α^−^ Th1 cells was elevated (Fig. 8C), and the proportion of VLA-4α^+^ Th1 cells to total Th1 cells was decreased in ES-DC-treated mice (Fig. 8D). Finally, we found that the presence of ES-DCs reduced VLA-4α expression in Th1 cells *in vitro* (Fig. 8E). Taken together, these results suggest that ES-DCs limit the migration of Th1 cells via the regulation of VLA-4α, and reduce tissue damage in some autoimmune diseases.

## Discussion

We previously showed that genetically modified ES-DCs inhibit the onset of the EAE, a model of autoimmune disease induced by immunization with a specific myelin auto-antigen [22]. We now demonstrate that both genetically modified and non-modified ES-DCs prevented the onset of diabetes in NOD mice, a spontaneously occurring autoimmune disease caused by immunity against multiple islet antigens, as well as EAE.

Some studies reported that modulated BM-DCs or lymph node (LN)-derived DCs were intravenously or intraperitoneally administrated to NOD mice, and the injections via both routes were effective [15]–[17], [51], [52]. We previously found that i.p. transferred ES-DCs accumulated in the spleen and mesenteric LNs [23], and a previous study reported the proliferation of splenocytes in response to islet-associated auto-antigens in 4-week old prediabetic female NOD mice [40], [53]. Therefore, although we examined the effect of i.p. injection of ES-DCs in the current study, i.v. therapy with ES-DCs may also exert the preventive effect.

In the current study, the disease-preventive effect of ES-DCs was accompanied with the reduction of numbers and frequencies of Th1 and Th17 cells in the spleen, but no effect on number of Treg cells was observed. The pathogenesis of diabetes in NOD mice is strongly linked to Th1 cells, and thus the treatment with ES-DCs may have effectively protected against islet β-cell destruction through the direct inhibition of pathogenic T cells.

However, even NOD mice injected five times with ES-DCs developed diabetes at around 2 years of age (data not shown), indicating that while ES-DCs induce long-term protection from diabetes, they do not completely extinguish auto-reactive T cells. Therefore, periodic and multiple administrations of ES-DCs are required for long-term protection from disease. Future clinical applications of DC treatment may require administration protocols to achieve lasting remission in human autoimmune diseases.

EAE is used as a mouse model of the human disease multiple sclerosis [54]. The pathogenesis of EAE involves the activation and invasion of myelin antigen-specific Th1 or Th17 cells into the central nervous system (CNS), leading to the expansion of resident microglia [55], [56]. In typical EAE, Th1 cells mainly penetrate the parenchyma of the spinal cord [57]. However, it has been reported that IFN-γ-deficient T cells induce atypical EAE in which cells infiltrate the cerebrum and brain stem but not the spinal cord [58]. Moreover, a higher ratio of Th17/Th1 cells has recently been found to be important for T cells to invade the cerebrum [59]. These results indicate that the initial EAE pathogenesis differs between the spinal cord and the brain in terms of types of auto-reactive T cells playing a major role.

We found that ES-DCs mainly regulated the differentiation and proliferation of Th1 cells, and that their capacity to regulate Th17 cells was less potent. Therefore, treatment with ES-DCs was more effective in decreasing infiltrating cells, especially Th1 cells into the spinal cord in typical EAE. In addition, difficulties in regulating Th17 cells may explain why clinical symptoms of EAE worsened slightly after ES-DC-treatment. The injection of ES-DCs also resulted in a decrease of CD11b^+^ myeloid cells in the spinal cord in the late phase of EAE. As blood-derived myeloid cells, including monocytes, are key for EAE progression [60], it is conceivable that the decrease in CD11b^+^ myeloid cells may also contributed to the development of clinical symptom.

Next, we discuss where i.v. administrated ES-DCs performed their therapeutic function. We chose i.v. but not i.p. injection to administrate ES-DCs for the inhibition of EAE. We previously observed that i.p. injection of non-genetically modified ES-DCs were not effective in prevention of EAE, although we used an ES cell line different from currently used one [22]. As above described, i.p. injected ES-DCs were mainly localized in the spleen and mesenteric LNs. In the experimental schedule of the present study, we planned to administer ES-DCs at day 10 after EAE-induction when the inflammation in the CNS would have already started. Therefore, we considered that i.v. rather than i.p. administration of ES-DCs would be more effective to achieve therapeutic effect, because i.v. injection of ES-DCs were expected to result in more efficient migration into the spinal cord and encounter with pathogenic T cells as compared with the case of i.p. injection. Actually, we detected a certain fraction of i.v. transferred ES-DCs in the spinal cord, and the proliferation of spinal cord-infiltrating Th1 cells was decreased by the administration of ES-DCs. These results suggest that ES-DCs contributes, at least in part, to the suppression of Th1 cell response in the CNS. Further investigations are required to identify the key molecules that enable ES-DCs to migrate into the affected organ.

A certain fraction of injected ES-DCs was detected also in the lungs. Distribution of ES-DCs to lungs was significantly decreased under inflammatory condition in comparison to that under steady-state condition, and the absolute number of lung-localized ES-DC under inflammatory conditions were also slightly lower (data not shown). These results may be attributed to compositive causes; decreased percentage due to dilution resulting from the infiltration of other immune cells including pathogenic T cells in the lungs, and absolute decrease resulting from the migration of ES-DCs into the affected organ.

Although the detection of ES-DCs in the lung is a natural transition as venous blood flow to the heart drains into the lungs through the pulmonary artery, the lung is also of great importance in autoimmunity. Pathogenic T cells travel from lymphoid tissues through the bloodstream to the lung where they acquire homing molecules to migrate to the target organ [48], [61]. Based on our findings that CD4^+^ T cells in the spinal cord were decreased in ES-DC-treated mice, we considered the possibility that ES-DCs down-modulate the expression of homing molecules on CD4^+^ T cells in the lung. We also observed a lower expression of VLA-4α on Th1 cells in ES-DC-treated mice. VLA-4α is a representative molecule for lymphocyte homing to the CNS. Indeed, the anti-VLA-4α antibody natalizumab was previously shown to impede the interaction of pathogenic T cells with VCAM-1 on endothelial cells, and to prevent the development of EAE and diabetes in mice [48], [62]. It is also effective in humans against multiple sclerosis and Crohn's disease [63], [64]. Therefore, the down-modulation of VLA-4α on Th1 cells by ES-DCs in the present study may have contributed to the suppression of the autoimmune disease. However, the key mediator of ES-DCs that inhibited VLA-4α on Th1 cells was not obvious, and further investigations are required.

Intrinsic immune regulatory function of ES-DCs seems to be limited to inhibition of Th1 response. Additional immune regulatory functions such as the inhibition of Th17 response or the promotion of Treg cells would enhance the capacity of ES-DCs to control autoimmunity. To this end, we have established a strategy for the genetic modification of ES-/iPS-DCs and macrophages [19]–[22]. Moreover, we previously reported that genetically modified ES-DCs expressing TRAIL induced the proliferation of Treg cells [65]. In the current study, we utilized genetically modified ES-DCs for the treatment of NOD mice. Future work will require the generation of ES-/iPS-DCs with greater immunoregulatory effects, and a means for selecting effective immunoregulatory molecules. However, if treatment with ES-DCs causes systemic immune suppression, it is same as existing immune suppressive drugs and ES-DCs will have no value in the treatment of autoimmune diseases. Herein, the T-cell dependent immune response to a foreign antigen were maintained, and ES-DCs may offer advantages against existing immune suppressive drugs.

In conclusion, we demonstrated the intrinsic function of ES-DCs to restore and maintain immune tolerance, leading to the prevention or improvement of autoimmune disease. Considering the recent advances in human iPS cell-related technologies, pluripotent stem cell-derived dendritic cells may be a promising means for the treatment of autoimmune diseases.

## Supporting Information

S1 Figure Surface phenotypes of NOD and B6 ES cell-derived DCs. (A–B) Flow cytometry analysis of indicated surface marker expression in (A) NOD-ES-DCs and (B) B6-ES-DCs. Staining pattern with specific antibodies (closed areas) and isotype-matched controls (gray lines) is shown. Data (mean ± SD) are representative of three independent experiments.
(TIF)

S2 Figure Genetic modification of ES-DCs. (A) The structure of pCAG-insulin-IPuro, pCAG-GAD65-IPuro, and pCAG-TRAIL-INeo. (B–C) Staining pattern with specific antibodies (closed areas) and isotype-matched controls (gray lines) is shown. (B) The expression of mutant human invariant chain (hCD74) bearing insulin peptide and HA-tag was examined using intracellular staining. (C) The expression of TRAIL on ES-DCs or genetically modified ES-DCs. (D) Functional expression of TRAIL in genetically modified ES-DCs was analyzed based on cytotoxicity against L929 cells. ^51^Cr-labeled L929 cells (5×10^3^) were incubated with ES-DCs, ES-DC-TRAIL, ES-DC-TRAIL/insulin, or ES-DC-TRAIL/GAD65 as effector cells at the indicated E:T ratio for 12 h. After incubation, cytolysis of target cells was quantified by measuring radioactivity in the supernatants. Results are expressed as mean specific lysis of triplicate assays. Data (mean ± SD) are representative of three independent experiments.
(TIF)
