# Supplementary figures and images for: Suppression of Th1-Mediated Autoimmunity by Embryonic Stem Cell-Derived Dendritic Cells
Source: PLoS One. 2014 Dec 18;9(12):e115198. doi: 10.1371/journal.pone.0115198 (PMC4270741; doi:10.1371/journal.pone.0115198)

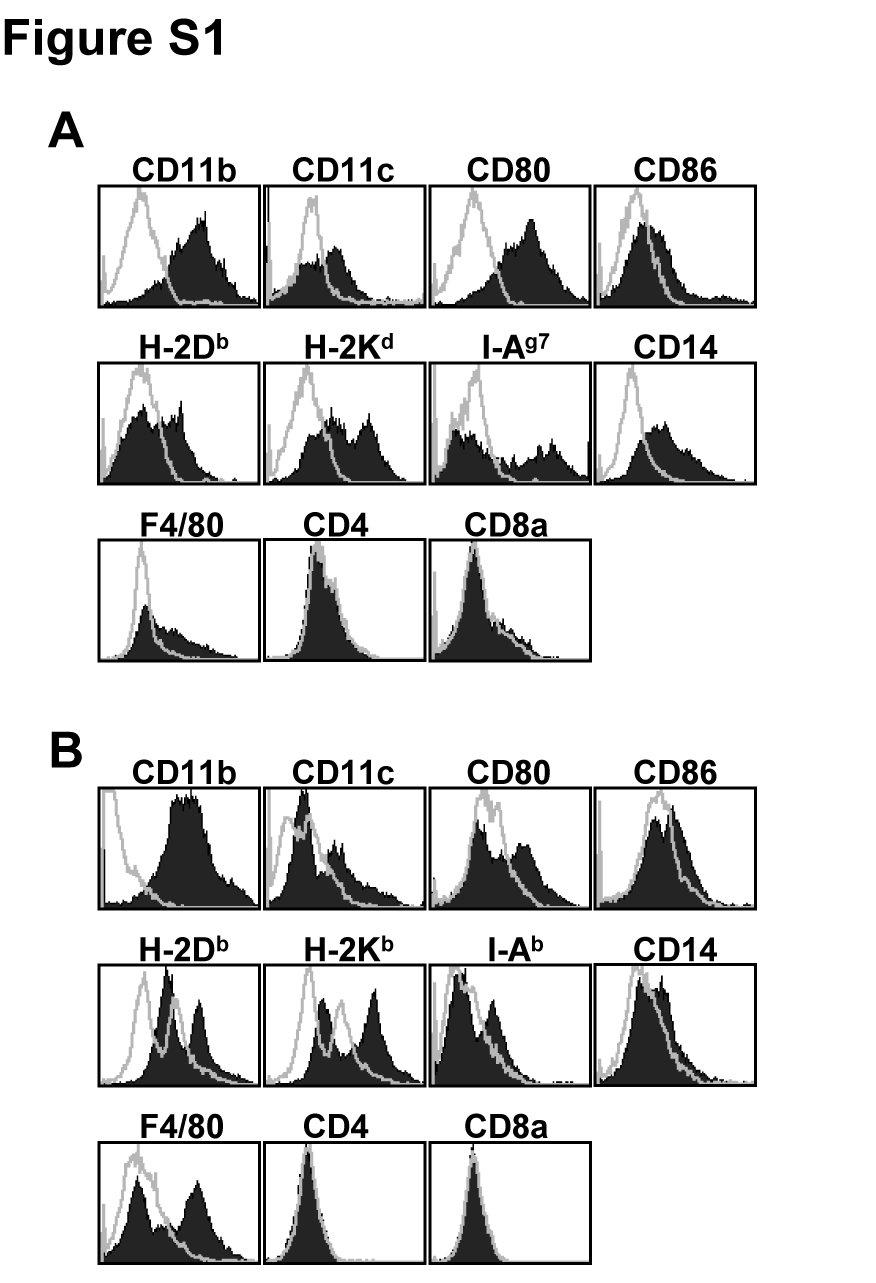

Supplement: S1 Figure — Surface phenotypes of NOD and B6 ES cell-derived DCs. (A–B) Flow cytometry analysis of indicated surface marker expression in (A) NOD-ES-DCs and (B) B6-ES-DCs. Staining pattern with specific antibodies (closed areas) and isotype-matched controls (gray lines) is shown. Data (mean ± SD) are representative of three independent experiments. (TIF) [file pone.0115198.s001.tif]

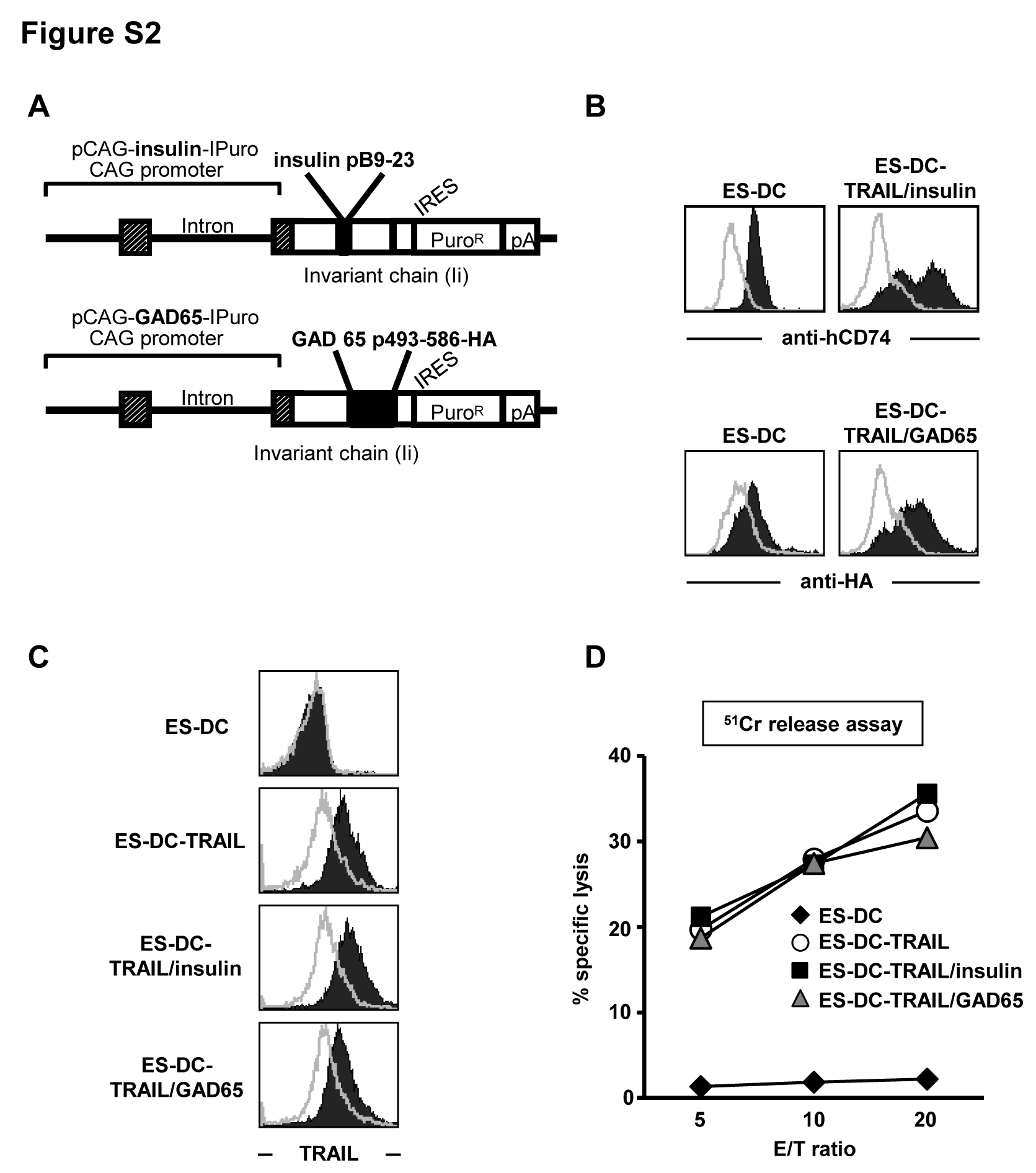

Supplement: S2 Figure — Genetic modification of ES-DCs. (A) The structure of pCAG-insulin-IPuro, pCAG-GAD65-IPuro, and pCAG-TRAIL-INeo. (B–C) Staining pattern with specific antibodies (closed areas) and isotype-matched controls (gray lines) is shown. (B) The expression of mutant human invariant chain (hCD74) bearing insulin peptide and HA-tag was examined using intracellular staining. (C) The expression of TRAIL on ES-DCs or genetically modified ES-DCs. (D) Functional expression of TRAIL in genetically modified ES-DCs was analyzed based on cytotoxicity against L929 cells. 51Cr-labeled L929 cells (5×10^3^) were incubated with ES-DCs, ES-DC-TRAIL, ES-DC-TRAIL/insulin, or ES-DC-TRAIL/GAD65 as effector cells at the indicated E:T ratio for 12 h. After incubation, cytolysis of target cells was quantified by measuring radioactivity in the supernatants. Results are expressed as mean specific lysis of triplicate assays. Data (mean ± SD) are representative of three independent experiments. (TIF) [file pone.0115198.s002.tif]
